# Construction of a 7-fold BAC library and cytogenetic mapping of 10 genes in the giant panda (*Ailuropoda melanoleuca*)

**DOI:** 10.1186/1471-2164-7-294

**Published:** 2006-11-17

**Authors:** Wei Liu, Yonghui Zhao, Zhaoliang Liu, Ying Zhang, Zhengxing Lian, Ning Li

**Affiliations:** 1 State Key Laboratory for Agro-Biotechnology, China Agricultural University, Beijing 100094, China; 2 College of Animal Science and Technology, China Agricultural University, Beijing 100094, China

## Abstract

**Background:**

The giant panda, one of the most primitive carnivores, is an endangered animal. Although it has been the subject of many interesting studies during recent years, little is known about its genome. In order to promote research on this genome, a bacterial artificial chromosome (BAC) library of the giant panda was constructed in this study.

**Results:**

This BAC library contains 198,844 clones with an average insert size of 108 kb, which represents approximately seven equivalents of the giant panda haploid genome. Screening the library with 15 genes and 8 microsatellite markers demonstrates that it is representative and has good genome coverage. Furthermore, ten BAC clones harbouring *AGXT, GHR, FSHR, IRBP, SOX14, TTR, BDNF, NT-4, LH *and *ZFX1 *were mapped to 8 pairs of giant panda chromosomes by fluorescence in situ hybridization (FISH).

**Conclusion:**

This is the first large-insert genomic DNA library for the giant panda, and will contribute to understanding this endangered species in the areas of genome sequencing, physical mapping, gene cloning and comparative genomic studies. We also identified the physical locations of ten genes on their relative chromosomes by FISH, providing a preliminary framework for further development of a high resolution cytogenetic map of the giant panda.

## Background

The giant panda is an endangered animal with a restricted habitat in South-western China. A survey revealed that only about 1,600 individuals remain in the wild [1]. The highly specialized reproductive behaviour [2] and low fertility make it difficult to increase their numbers quickly by breeding in captivity. In recent years, strenuous efforts have been made to protect this animal and considerable knowledge of its physiology, biochemistry, genetic diversity and ecology has been achieved, but research on the giant panda genome is still rare. So far only a few genes have been cloned and two have been mapped to specific giant panda chromosomes [3,4].

A bacterial artificial chromosome (BAC) library is a powerful tool for studying genomes. Compared with yeast artificial chromosome (YAC) clones, BAC clones have many advantages such as high stability, easy manipulation and rare chimerism [5,6]. BAC libraries of human [7] and major livestock [8-11] have been constructed and widely used, but no such large-insert genomic DNA library has been reported to date for the giant panda.

We have constructed and characterized a seven-genome equivalent BAC library of the giant panda, and located ten genes to specific chromosomes by FISH using their BAC clones as probes.

## Results

### BAC library construction

The BAC library was constructed by cloning genomic DNA isolated from the white blood cells of a male giant panda, and partially digested with *Bam*HI/*Hin*dIII, into the vector pBeloBAC11. It contains 198,844 clones, which were deposited in 518 384-well plates. Among these 198,844 clones, 145920 were from *Bam*HI digestion and 52924 from *Hin*dIII digestion.

### Insert size distribution

**Figure 1:**
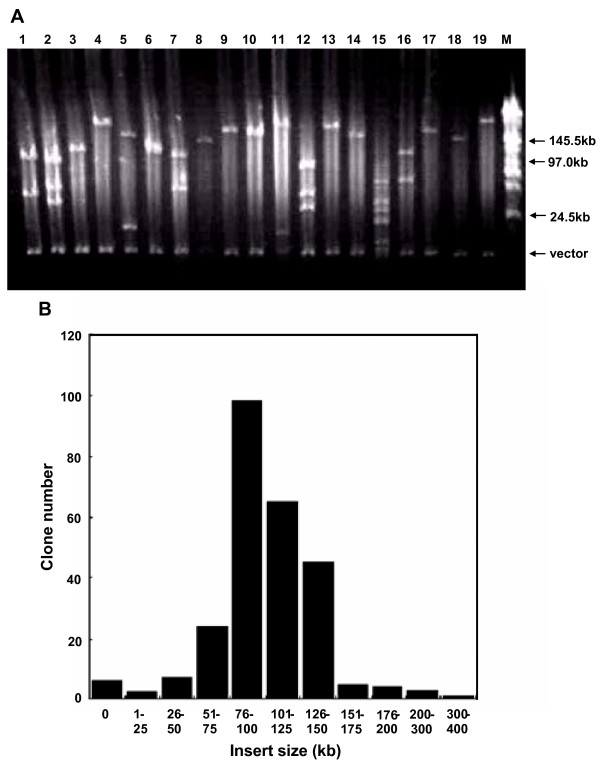
**Characterization of the giant panda BAC library**. **A**. Analysis of the size of BAC clones by PFG electrophoresis. M: Mid range Marker II for PFGE (Bio-labs, New England). Lanes 1–19: *Not*I digested DNA of randomly picked recombinant BAC clones. PFGE conditions were set with Bio-rad CHEF III: 6.0 v/cm for 12 h, linear pulse time ramping from 0.1 to 40 s. **B**. BAC insert size distribution in the library. Insert sizes were determined from 261 BAC clones. The horizontal axis shows the size range in kb while the vertical axis displays the number of clones corresponding to each size range. Insert sizes are reported in a cumulative histogram.

To evaluate the average size of inserted fragments in the library, DNAs were prepared from 261 randomly selected clones, cleaved with *Not*I and subjected to pulse-field gel electrophoresis with midrange PFG Marker II (New England Biolabs, USA). The insert size distribution is shown in Figure 1. The average insert size of these 261 clones is 108 kb, indicating that the library represents a 7-fold coverage of the giant panda haploid genome. Among the 261 clones, only 6 had no insert, suggesting that the percentage of non-recombination in the library is about 2.3%. Approximately 53% of the clones contain inserts larger than 100 kb.

### BAC library screening

To assess the quality of this library further, 8 microsatellite markers were used for screening. The number of positive superpools varied from 1 to 10 with an average of 4.6 (Table 1). At the same time, 15 giant panda genes (*ACTIVIN, AGXT, BDNF, FSH, FSHR, IRBP, GHR, GpH, GDNF, NT-4, LH, SOX14, TTR, ZFX1 *and *ZFX2*) were also screened in the library using PCR. As shown in Table 2, the number of positive BAC clones varied from 2 to 8 with an average of 4.7. The actual number of clones obtained was slightly lower than was calculated theoretically, but at least 2 positive BACs for each of the genes were identified from the library.

### Gene mapping by FISH

**Figure 2:**
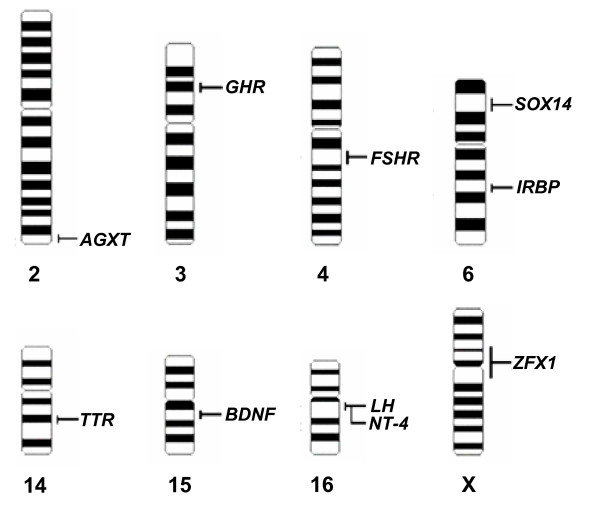
**Physical locations of ten genes on G-banded ideogram of giant panda**. The G-banded ideogram was drawn with software Video TesT-karyotype3.1 (Video TesT Ltd, Russia, 2003). The names of the bands have not been listed because no international standard karyotype has yet been established for the giant panda.

To estimate the fraction of chimeric clones in the BAC library, and furthermore to map some of the genes, a FISH approach was used. Ten BAC clones, containing *AGXT, GHR, FSHR, IRBP, SOX14, TTR, BDNF, NT-4, LH *and *ZFX1 *respectively, were mapped to 8 pairs of giant panda chromosomes. The specific locations of the 10 functional genes on the G-banded ideogram are listed in Figure 2. The band names of each chromosome are not given because no international standard karyotype has yet been established for the giant panda. The order of chromosomes was identified according to a previous study [12] by analyzing the size, G-band and centromere location. Fifteen metaphases were analyzed to map each gene.

## Discussion

We have successfully constructed a giant panda BAC library, which is large-insert, deep-coverage and publicly available. One obstacle to genomic and molecular biology research on the giant panda is the difficulty of collecting samples. This BAC library will partially solve the problem.

BAC clones can be used as valuable probes for cytogenetic mapping by FISH. In this study, we successfully mapped 10 genes to giant panda chromosomes using their BAC clones as probes. Thus, the number of molecular markers on giant panda chromosomes could be increased to 12. However, a major obstacle to this study in FISH mapping is the preparation of chromosome samples. Giant panda fibroblast cells cultured in our laboratory grew too slowly to provide sufficient high-quality chromosome samples. Therefore, only ten genes were mapped to chromosomes, though we had prepared for localizing fifteen genes by using the relative BAC clones.

The homologous relationship of chromosomes between human and giant panda has been established on the basis of comparative painting studies [13], so we deduced the approximate chromosome locations of the ten giant panda genes (Table 3) according to their human chromosome homologues [14]. Six genes, *AGXT, GHR*, *FSHR, IRBP, SOX14 *and *TTR*, were mapped to their expected chromosomes by FISH (Table 3), confirming the homologies of six pairs of chromosomes between giant panda and human, and identifying more precisely the regions of these chromosomes that are conserved between the two species.

The signals of the BAC clone harbouring *ZFX1 *were visualized both around the centromere of chromosome X and at the terminal of chromosome Y. Since *ZFX1 *is located on chromosome X in almost all mammals, the specific signal at the terminal of chromosome Y may be because the BAC clone contains a fragment that is homologous between chromosomes X and Y, or because it is a chimeric clone.

**Figure 3:**
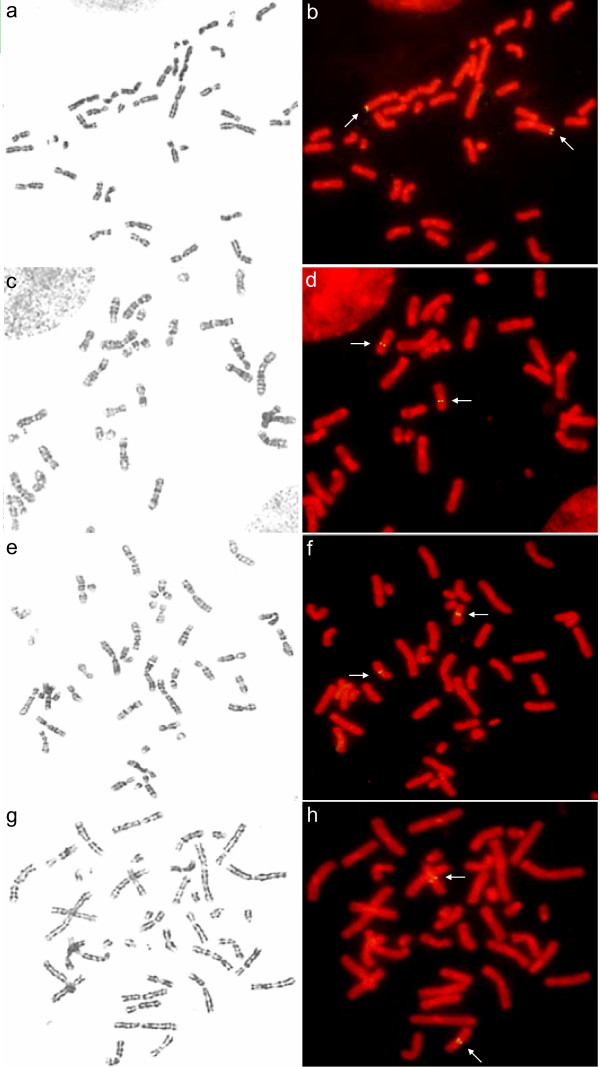
**Representative scheme of chromosome locations of *AGXT *(a+b), *BDNF *(c+d), *LH *(e+f), and *NT-4 *(g+h) in giant panda identified by FISH**. a, c, e, g show G-banded metaphase chromosomes spread before FISH; b, d, f, h show the same chromosomes spread after FISH. Arrow shows the specific signals.

*LH *and *NT-4 *were both mapped on the same band of AME16q (Figures 2, 3), and these two genes are also close in the human genome (Table 3). This suggests that they belong to a conserved genome block. *BDNF *was mapped to chromosome 15q; its homologue in human is located at chromosome 11p14.1, which indicates the conserved syntenic homology between AME15q and HSA11p. However, the mapping results for these three genes are not consistent with the giant panda-human comparative painting data (Table 3) owing to the problem of identifying chromosome order. In the giant panda, the sizes and morphologies of chromosomes 12 to 16 are so similar that the same chromosome has been designated different chromosome orders in different studies [12,13,15]. This problem will be solved when more markers are mapped to these chromosomes.

## Conclusion

The first representative giant panda genomic BAC library has been constructed, covering about seven equivalents of the giant panda genome. This BAC library will serve as a valuable resource for genomic research on the endangered species. We also mapped 10 genes to their relative chromosomes by FISH using their BAC clones as probes, and this enriched the giant panda cytogentic map.

## Methods

### Preparation of vector and HMW DNA

The BAC vector pBeloBAC11 was purified by CsCl-ethidium bromide density gradient centrifugation, digested with the appropriate amount of *Hin*dIII or *Bam*HI (New England Biolabs, USA) and treated with shrimp alkaline phosphatase (USB, USA). Linear vectors were recovered by electroelution as described by Osoegawa et al. [16]. A DNA plug was prepared as described by Birren et al. [17] from the white blood cells of a male giant panda and partially digested using 5 units of *Hin*dIII or *Bam*HI per μg DNA at 37°C for 45 min. Size-fractionation was performed in a CHEF III apparatus (Bio-Rad, USA) and DNAs were separated in three stages as described by Osoegawa et al. [16]. DNA fragments with range of 150–250 kb were recovered by electroelution.

### Ligation and transformation

At an approximately 1:10 molar ratio of insert: vector, 150 kb–250 kb DNA fragments were ligated to 30 ng of linearized pBeloBAC11 vector in a 120 μl total volume and incubated at 12°C for 24–48 h. The ligation mixture was then dialyzed on a microdialysis filter (0.025 μm pore size; Millipore) against 0.5 × TE for 40 min. Two microlitres of the ligation product was used to transform 20 μl ElectroMAX DH10B competent cells (Gibico, USA) by electroporation on a Gene Pulser II (Bio-rad). The transformation parameters were as follows: resistance of pulse controller plus, 100 Ω; voltage gradient, 18 kv/cm; capacitance, 25 μF. In total, 87 electroporations were performed to make the panda *Hin*dIII and *Bam*HI library. Transformed cells were frozen at -70°C prior to colony picking.

### Library pooling

The frozen stocks of the primary clones were recovered and spread on large selection plates. Colonies with diameters >1.5 mm were picked manually and incubated overnight at 37°C in 96 deep well plates containing LB medium supplemented with 10% glycerol and 12.5 μg/ml chloramphenicol. Using MultiPROBE^® ^II automated liquid handling systems (PerkinElmer, USA), 80 μl of cultures from four 96 plates were transferred to 384 well plates and stored at -70°C. To establish the two-step PCR screening systems, the library was divided into 26 superpools and one superpool comprised 20 384-well plates. Cultures from every superpool were combined to make superpool DNA for the first step PCR screening. In each superpool, cultures from each plate (384 clones), row (24 clones × 20 plates) and column (16 clones × 20 plates) were combined respectively to make DNA for the second step screening. The combined cultures were centrifuged and the bacterial pellet was suspended in TE then boiled in a microwave oven. After centrifugation, the supernatants containing the pooled DNA were transferred and stored in 96 well plates. The stock DNA was diluted 20 times to the working concentration for PCR screening.

### Estimation of average insert size

Two hundred and sixty-one recombinant BAC clones were randomly picked and grown in LB medium overnight at 37°C. DNA was prepared by alkaline lysis and digested with *Not*I, then separated by PFG electrophoresis. PFGE conditions were set with Bio-Rad CHEF III: 6.0 v/cm for 12 h, linear pulse time ramping from 0.1 to 40 s.

### BAC library screening

Primers for each gene and some microsatellite markers were designed according to the giant panda DNA sequences published in NCBI, and the other microsatellite markers were designed according to the sequences from our laboratory (A204, A066 and B043). BAC screening was performed by two-step PCR (superpools PCR and 4D-PCR) [see Additional file 1]. Positive BAC clones were confirmed by sequencing of PCR products.

### Fluorescence in situ hybridization

For FISH analysis, chromosomes were prepared from a male giant panda fibroblast using a standard cytogenetic protocol. The FISH method was modified from the previous study [18]. Briefly, chromosomes were performed G-bands and well-banded metaphases were photographed. The BAC clones of 10 functional genes (Table 3) were used as probes and were labelled by nick translation with biotin-14-dATP (Invitrogen, Carlsbad, CA92008, USA). The denatured probes were dissolved in hybridization solution to a final concentration of 50 ng/μl and prehybridized for 40 min at 37°C. Hybridization was performed for 17 hours at 37°C in a humid chamber. Probes were detected with FITC-conjugated avidin (Vector, Burlingame, CA94010, USA) and signals were amplified by biotinylated anti-avidin (Vector). Chromosomes were counterstained with 0.5 μg/ml propidium iodide. Images were obtained with an epifluorescence microscope equipped with a DP70 CCD camera (Olympus, Japan). G-banded ideograms were drawn with Video TesT-karyotype3.1 software (Video TesT Ltd, Russia, 2003).

## Authors' contributions

WL constructed the BAC library and drafted the manuscript with YHZ, ZLL and NL. YHZ participated in the gene screening and FISH mapping. ZLL and YZ contributed to the BAC library pooling and screening. ZXL and NL conceived the project and supervised its execution. All authors read and approved the final manuscript.

## Supplementary Material

Additional File 1 Scheme representations of BAC library and PCR screening systems. Detailed method of two-step screening of the BAC library (superpools PCR and 4D-PCR) is shown. Click here for file

## Figures and Tables

**Table 1:** Summary of screening results of the giant panda BAC library by PCR with primers for microsatellites

Microsatellite Sequence Name	Primer (5'-3')	T_a _(°C)	Product length (bp)	Positive superpool number
A204	F- CCTGCCCTATGGATTTCAGA R-GGTCCATATCATGGTCTAAATGA	63	321	10
A066	F- GATTATCCGTTGGCTTTGGA R- CGTGGTTGTCTCCACACTTG	60	337	3
B043	F- GACCCTGTGGCTCTGTGACT R- GCGAGGGAAGGAAGAGAAAC	60	310	6
GPMC-1	F- AGAGAGAGAGCGAGCACGAG R- AGCCACGAAACAGAGAGGAA	63	207	1
GPMC-21	F- GAATGGTATGCCTGGGTGAC R- AACACAAGCGGGGGAACT	63	150	6
GPMC-40	F- TCCCTCAGCACCTTAACACC R- TACTGGCCCAGGACACTAGC	58	214	4
GPMC-52	F- CCGTCTCCAAGGTAACCTGA R- AGAGATTGCTAGGCGGACAA	60	190	3
GPMC-104	F- TCTCCATCAGGTTACCACTGC R- CTGCGTGCCTCAGTCAGTTA	60	261	4

**Table 2:** Summary of screening results of the giant panda BAC library by PCR with primers for functional genes

Gene	Genebank accession No.	Primers (5'-3')	T_a _(°C)	PCR product size (bp)	Positive clone number
***ACTIVIN***	AF350256	F- GGACATTGGCTGGAATGACT R- GACATGGGTCTCAGCTTGGT	54	207	4
***AGXT***	AJ539311	F- CTGTTTTTCATTGACTGGACTCTG R- TCTCATCCATTATCTGGGGG	58	539	4
***GHR***	AF367203	F- CCTCTGGGGGAAAAAAAGTT R- TCATCATCCTTTGCTCCAAG	56	412	8
***FSH***	AF448454	F- TGTTGCTGGAGAGCAATCTG R- TACGCCAGCTCTTTGAAGGT	54	197	7
***FSHR***	AF415208	F- GCCAAAGGATTTGACACGAT R- TGGTGACCAGGATCATCAGA	56	202	6
***GpH***	AF448453	F- GCCCAGAATGCAAGCTAAAG R- ACTGTGGCCTTGGTAAATGC	54	190	5
***SOX14***	AF016377	F- AACCCCAAGATGCACAACTC R- ACGTAGCCTCTTGGCCTCAC	54	108	6
***IRBP***	AY303836	F- CCAGGAAGTGGTGAGCAAGC R- CGGTGAGGAAGAAGTCGGAC	66	419	4
***TTR***	AF039738	F- TCTGCAGAGCTCACTGAACC R- TTCGATTAGCTGAGGGGAAA	54	205	4
***BDNF***	U56638	F- TCTGGAGAGCGTGAATGGG R- CGATACAGGGACTTTTTCAAGG	62	394	3
***GDNF***	AF516767	F- TGAAAAGGTCACCCGAGAAG R- AGCCGCTGCAGTACCTAAAA	54	219	5
***NT-4***	AY189135	F- AGCGAGACAGCACCAGCAA R- TCAGACACCCAGTGCCTCC	64	263	5
***LH***	AF448455	F- GCTGTGGCTGCTGCTGAAC R- CCACAGGGAAGGAGACCATG	58	313	2
***ZFX1***	AF016379	F- TCGGTTCACACGAAGGACTAC R- TGATACACCTTCCTGCCACTG	59	284	4
***ZFX2***	AF016380	F- GGTGCAGCAACATGCTCTTA R- CCACGTGTTTCTTGAGTTCG	54	188	4

**Table 3:** Chromosomal locations of ten genes in giant panda and of the homologous genes in human

Gene	Location in giant panda chromosome (AME)	Location in human chromosome (HSA)	Expected location in giant panda chromosome (AME)*	Clone Address**
***AGXT***	2q	2q37.3	2	221E23
***GHR***	3p	5p12	3	323D6
***FSHR***	4q	2p16.3	4	331F20
***SOX14***	6p	3q22.3	6	168E8
***IRBP***	6q	10q11.22	6	243M3
***TTR***	14q	18q12.1	14	29N4
***BDNF***	15q	11p14.1	16	212F3
***NT-4***	16q	19q13.33	12	229L4
***LH***	16q	19q13.33	12	64A12
***ZFX1***	X	Xp22.11	X	403N3

* The expected chromosome locations of the ten genes in the giant panda according to human-giant panda comparison.

** BAC clone address for FISH mapping.
