# Supplementary material for: Construction of a 7-fold BAC library and cytogenetic mapping of 10 genes in the giant panda (Ailuropoda melanoleuca)
Source: BMC Genomics. 2006 Nov 17;7:294. doi: 10.1186/1471-2164-7-294 (PMC1664575; doi:10.1186/1471-2164-7-294)
Supplement: Additional File 1 — Scheme representations of BAC library and PCR screening systems. Detailed method of two-step screening of the BAC library (superpools PCR and 4D-PCR) is shown. [file 1471-2164-7-294-S1.pdf]

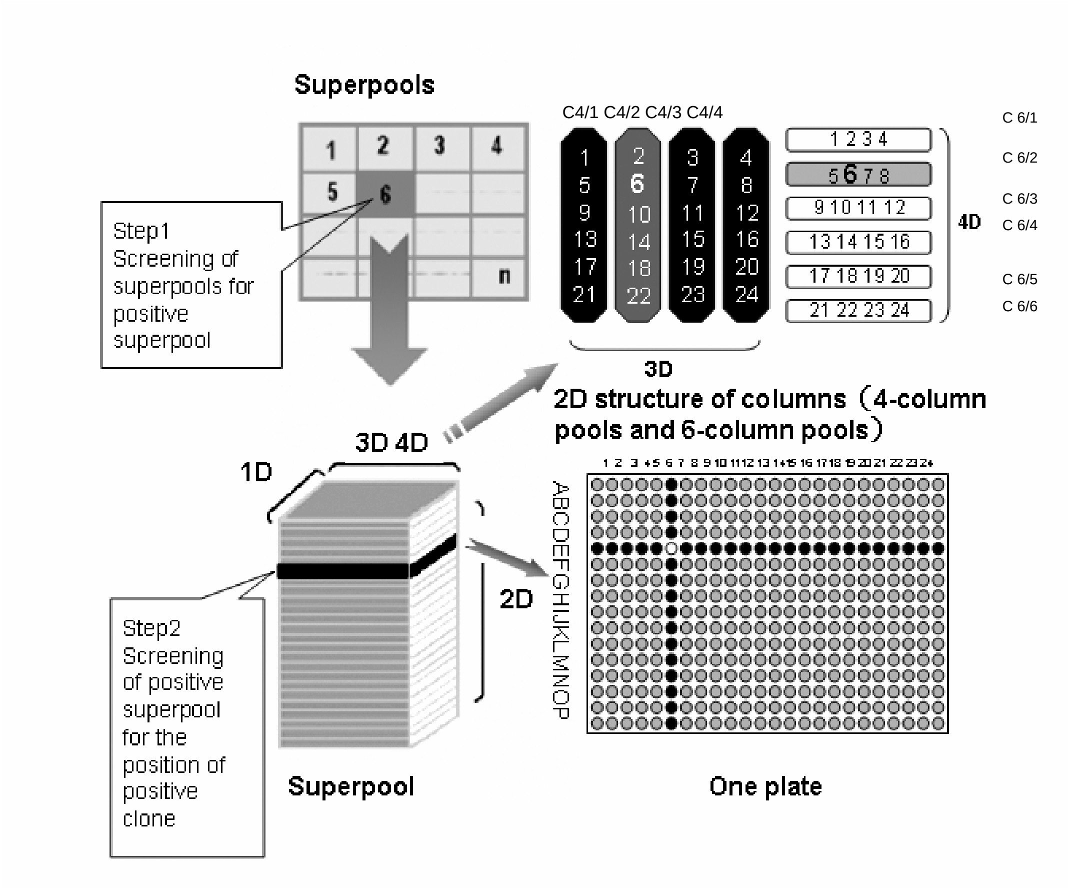

Additional file 1. Scheme representations of BAC library and PCR screening systems

Step 1, Screening against 26 superpools:

A positive signal was detected in the superpool 6 (plates PB 0101-0120).

Step 2, Screening of superpool 6 by 4D-PCR:

The conversion of the results to a specific clone was shown: Positive signals were identified in 1D, 2D, 3D and 4D (24 columns were organized into 10 pools in 2 dimensions); these consisted of E, p5, C4/2(3D) and C6/2(4D) respectively, indicating that row E, p5 and column 6 contained the specific BAC DNA. 2D-p5 was indicated on the plate PDB0105. Consequently, a BAC clone containing the specific sequence has been identified to be a clone of PDB0105 E6.
